# Annexin A6 Interacts with Newcastle Disease Virus Matrix Protein and Is Associated with Reduced Viral Replication in DF-1 Cells

**DOI:** 10.3390/v18101164

**Published:** 2026-10-20

**Authors:** Haixu Xu, Yu Chen, Shunlin Hu, Xiufan Liu

**Affiliations:** 1 School of Food and Biology Engineering, Xuzhou University of Technology, Xuzhou 221000, China; xhx@xzit.edu.cn; 2 Animal Infectious Disease Laboratory, College of Veterinary Medicine, Yangzhou University, Yangzhou 225012, China; biochenyu@hotmail.com; 3 Jiangsu Co-Innovation Center for Prevention and Control of Important Animal Infectious Diseases and Zoonosis, Yangzhou University, Yangzhou 225012, China; 4 Jiangsu Key Laboratory of Zoonosis, Yangzhou University, Yangzhou 225012, China

**Keywords:** Newcastle disease virus (NDV), matrix (M) protein, annexin A6 (AnxA6), viral replication, host-pathogen interaction

## Abstract

Newcastle disease virus (NDV) incurs substantial economic losses within the global poultry industry. The matrix (M) protein of NDV is integral to viral replication. In this study, we examined the interaction between the NDV M protein and annexin A6 (AnxA6) in DF-1 cells. Our findings revealed that AnxA6 associates with the C-terminus of the NDV M protein, predominantly within the cytoplasm. Functional analyses indicated that the knockdown of AnxA6 expression significantly enhanced NDV replication, whereas the overexpression of AnxA6 inhibited viral replication. Taken together, these findings suggest that AnxA6 may function as a negative regulator of NDV replication in this cell culture model, potentially through its interaction with the M protein. This study offers new insights into the intricate relationship between viral proteins and host factors in NDV infection and suggests that AnxA6 may serve as a potential target for further investigation. Further research is necessary to elucidate the detailed mechanisms governing the AnxA6-M protein interaction.

## 1. Introduction

Newcastle disease virus (NDV), a member of the Paramyxoviridae family, is one of the most fatal pathogens affecting global poultry production, causing large economic losses [1,2,3]. This enveloped virus possesses a monopartite, single-stranded negative-sense RNA genome of approximately 15.1 kb, which encodes six structural proteins arranged sequentially as 3′-NP-P-M-F-HN-L-5′ [4,5]. Of particular significance is the matrix (M) protein, a 40 -kDa structural component anchored to the inner leaflet of the viral envelope through hydrophobic interactions [6,7]. Studies have demonstrated that the M protein orchestrates multiple phases of the viral life cycle, including (1) nuclear-cytoplasmic trafficking of viral components [8,9], (2) modulation of viral RNA synthesis through polymerase complex interactions [10,11,12], (3) assembly of progeny virions via membrane curvature induction [6,7,13], and (4) coordinated budding through the endosomal sorting complex required for transport (ESCRT) machinery recruitment [14,15,16].

In recent years, studies have elucidated the interactions between NDV M proteins and host proteins, indicating their essential role in modulating NDV infection [17,18,19,20,21,22]. For example, Duan et al. identified the nucleolar phosphoprotein B23 as an interacting partner of the M protein, showing that this interaction affects nucleolar localization and enhances NDV replication and pathogenicity [4,23,24]. Furthermore, interactions with host proteins such as chicken bromodomain containing protein 2 (chBRD2) and importin β1 promote NDV replication by regulating gene expression and viral RNA synthesis [17,18,25]. Moreover, interactions between the M protein and cellular components such as actin and members of ESCRT machinery are essential for accurate assembly and release of NDV particles [21,26]. These interactions highlight the intricate relationship between the NDV-M protein and host factors in viral replication and pathogenicity. Additionally, studies have revealed the impact of the NDV M protein on host immune responses, with interactions observed between M proteins and host proteins involved in immune regulation [27,28,29]. Notably, interactions between the M protein and ANP32B or viperin are linked to the immune manipulation or evasion of NDV, respectively [22,30,31,32].

Annexins are a family of closely related calcium- and membrane-binding proteins that are expressed in most eukaryotic cell types [33,34,35]. Despite their structural and biochemical similarities, annexins have diverse functions including vesicle trafficking, cell division, apoptosis, calcium signaling, and growth regulation [36,37,38]. Annexin A6 (AnxA6) is an unique member of the annexin family and contains eight rather than four annexin repeats that link Ca^2+^ signaling and membrane functions [39]. AnxA6 interacts with the μ1 and μ2 subunits of the AP1-clathrin complex of the clathrin-mediated endocytosis process, which is exploited by the influenza A virus (IAV) to enter the host cells [40,41]. Another study reported that AnxA6 interacts with the M2 ion channel protein cytoplasmic tail (CT) of the IAV and negatively modulates viral infection by affecting viral budding and release [33,39,42].

The structure of paramyxovirus matrix proteins has been revealed through cryo-electron microscopy studies, showing a highly organized protein architecture essential for viral assembly [43]. The M protein undergoes various post-translational modifications, including ubiquitination, which regulate its subcellular localization and function [8,9]. Recent studies have also highlighted the role of autophagy in NDV replication, with the virus modulating autophagic pathways to enhance its replication [44,45]. Furthermore, the M protein has been shown to interact with various cellular factors involved in the interferon response, contributing to viral immune evasion [46,47].

Considering the similarities in function and interactions with host proteins observed in other viruses, we hypothesized that the NDV M protein interacts with AnxA6 in host cells. To this end, GST pull-down and co-immunoprecipitation assays were performed on NDV-infected cells to identify this specific interaction. Moreover, we demonstrated that the C-terminus of the NDV-M protein is involved in M-AnxA6 interaction. Further characterization of the knockdown of AnxA6 or overexpression of AnxA6 in host cells revealed that AnxA6 negatively regulated NDV replication in DF-1 cells. Our findings suggested that this interaction negatively regulates NDV replication in vitro, providing insights into the complex interplay between viral proteins and host factors during viral infections.

## 2. Materials and Methods

### 2.1. Cells, Viruses, and Antibodies

The DF-1 chicken embryonic fibroblast cell line and HEK-293T human embryonic kidney cell line were obtained from the Cell Resource Center at the Shanghai Institutes for Biological Sciences, Chinese Academy of Sciences. A recombinant NDV strain, rZJ1GFP, engineered to express green fluorescent protein (GFP), was constructed in a prior study [48,49]. This virus underwent three rounds of plaque purification in DF-1 cells followed by a single round of amplification in specific pathogen-free (SPF) embryonated chicken eggs. The anti-FLAG antibody (F1804) was sourced from Sigma-Aldrich (Darmstadt, Germany), while the anti-c-Myc antibody (ab72468) was provided by Abcam (Cambridge, UK). Monoclonal antibodies targeting GFP (sc-9996) and β-actin (sc-1616-R) were purchased from Santa Cruz Biotechnology (Dallas, TX, USA). Additional primary antibodies, including rabbit anti-AnxA6 polyclonal antibody (sc-11388) and mouse anti-GST monoclonal antibody (sc-374171), were also acquired from Santa Cruz Biotechnology (Dallas, TX, USA). The rabbit anti-NDV M polyclonal antibody was kindly supplied by Dr. Chan Ding from the Shanghai Veterinary Research Institute, Shanghai, China. This antibody was generated using a purified recombinant NDV M protein (strain ZJ1) as the immunogen in rabbits. Its specificity for the NDV M protein was validated by Western blot analysis, showing a single band at the expected molecular weight (~40 kDa) in NDV-infected cell lysates and in cells transfected with M-expression plasmids, with no signal detected in uninfected or mock-transfected control cells. The anti-F and anti-HN antibodies used for immunofluorescence assays were polyclonal rabbit antibodies generated against the purified ectodomains of the F and HN proteins of NDV strain ZJ1, respectively (kindly provided by Dr. Chan Ding, Shanghai Veterinary Research Institute, Shanghai, China). Their specificity was confirmed by Western blot analysis, each detecting a single band corresponding to the expected molecular weight of the respective glycoprotein in NDV-infected cells but not in mock-infected controls.

### 2.2. Plasmids and Construction

The expression plasmids pCMV-Myc, pDsRed-monomer-N1, and pEGFP-C1 were acquired from Clontech (Mountain View, CA, USA), while pGEX-6p-1 and pET-32a (+) vectors were sourced from Invitrogen (Waltham, MA, USA). The chicken AnxA6 gene (chAnxA6; accession no. NM_204730.1) was amplified from DF-1 cell cDNA using specific primers: forward primer 5′-CGCGAATTCATGGCACCCAAAGGAAAGGT-3′ and reverse primer 5′-CGCCTCGAGCTAGTCGTCCCCCCCGCACA-3′. For the construction of Myc, Red, or GST epitope-tagged expression vectors, the PCR-amplified fragments were digested with restriction enzymes and subsequently cloned into pCMV-Myc, pDsRed-monomer-N1, and pGEX-6p-1, respectively. The full-length NDV M protein gene was amplified from the cDNA clone of the NDV strain Goose/China/ZJ1/2000 (ZJ1, GenBank: AF431744), digested with EcoR I-Sal I or EcoR I-Xho I, and ligated into pEGFP-C1 and pGEX-6p-1 to generate GFP or GST-tagged constructs. Truncated M protein mutants were created by cloning PCR-amplified fragments into the pET-His vector. The recombinant plasmids pCMV-Myc-M, pCMV-Myc-AnxA6, pEGFP-M, pDsRed-AnxA6, and pDsRed-M were constructed using standard molecular cloning techniques. All primers required for plasmid construction were synthesized by Sangon Biotech Company (Shanghai, China) and are detailed in Table 1. The integrity and accuracy of all recombinant plasmids were verified through PCR, restriction enzyme analysis, and DNA sequencing.

### 2.3. Cell Culture, Transfection, and Fluorescence Microscopy

The DF-1 chicken embryo fibroblast cell line and HEK-293T human embryonic kidney cells were maintained in Dulbecco’s modified Eagle’s medium (DMEM) enriched with 10% fetal bovine serum (FBS) and incubated at 37 °C in a humidified environment with 5% CO_2_. For transfection assays, HEK-293T cells were plated at a density of 5 × 10^4^ cells per well on glass coverslips in 12-well plates, aiming for approximately 60% confluency. Plasmid transfection was performed using FuGENE^®^ HD Transfection Reagent (Roche, Basel, Switzerland) following the manufacturer’s protocol [17,18]. After 24 h of transfection, cells expressing fluorescently tagged proteins were fixed with 4% paraformaldehyde for 20 min, permeabilized with 0.2% Triton X-100 for 5 min, and imaged using a Leica fluorescence microscope (Leica Microsystems GmbH, Wetzlar, Germany; model DMI4000B, equipped with a Leica DFC350FX digital camera). Images were captured at ×200 magnification under identical exposure settings for each fluorescent channel. Multiple representative fields were examined for each experimental condition to ensure consistent and reproducible fluorescence patterns. Nuclei were counterstained with DAPI (Sigma-Aldrich, Darmstadt, Germany) to facilitate visualization.

### 2.4. Protein Interaction Assays

In pull-down experiments, GST-M and GST-A6 recombinant proteins were expressed and purified from bacterial cultures transformed with pGEX6p-M and pGEX6p-A6 plasmids, respectively. DF-1 cells were lysed using ice-cold lysis buffer (Beyotime, Shanghai, China), and 1 mg of the resulting cell lysate was incubated with 10 µg of GST-M or GST protein bound to Glutathione -Sepharose beads at 4 °C for 3 h. After extensive washing with lysis buffer, the samples were separated by SDS-PAGE and analyzed for AnxA6 using a specific anti-A6 antibody. Similarly, a GST pull-down assay was performed using GST-A6 protein and GFP-M protein derived from HEK-293T cells transfected with pEGFP-M, following the same protocol.

For co-immunoprecipitation (co-IP) experiments, DF-1 cells seeded in six-well plates were co-transfected with plasmids encoding the full-length M protein, chAnxA6 protein, or their respective truncation mutants. At 36 h post-transfection, cells were washed with PBS and lysed using immunoprecipitation lysis buffer (Pierce, Rockford, IL, USA). The clarified supernatants were incubated overnight at 4 °C with either anti-GFP or anti-Myc antibodies. Immune complexes were then captured using protein A+G-Sepharose beads (Sigma-Aldrich, Darmstadt, Germany) for 3 h at 4 °C. After three washes with immunoprecipitation lysis buffer, the precipitated proteins were analyzed by Western blotting using anti-Myc or anti-GFP antibodies.

### 2.5. Immunofluorescence Analysis of Virus-Infected Cells

DF-1 cells were infected with NDV strain rZJ1GFP or rZJ1 at a multiplicity of infection (MOI) of 0.1 and analyzed via immunofluorescence at different hours post-infection (hpi). For the co-localization study of M protein and AnxA6 in virus-infected cells, cells were additionally fixed at 16 hpi to capture peak protein expression levels. At each time point, the cells were washed with PBS, fixed using 4% paraformaldehyde for 20 min, and washed three times with PBS. Permeabilization was achieved by incubating the cells with 0.2% Triton X-100 in PBS for 5 min. Following another PBS rinse, the cells were blocked with 10% fetal bovine serum (FBS) in PBS for 30 min and then incubated with anti-A6 and anti-M antibodies diluted in PBS containing 10% FBS for 1 h. After three additional PBS washes, the cells were incubated with Alexa Fluor 488-conjugated goat anti-mouse IgG and Alexa Fluor 594-conjugated goat anti-rabbit IgG antibodies (Invitrogen, Waltham, MA, USA) for 1 h. Nuclei were counterstained with DAPI, and the cells were imaged using a fluorescence microscope (Leica, Wetzlar, Germany). The acquired images were further processed using Adobe Photoshop software for analysis and presentation. All images were acquired under identical exposure and gain settings to allow direct comparison between experimental groups.

### 2.6. Small Interfering RNA (siRNA) Treatment and Virus Infection

Table 2 presents the sequences of three siRNA pairs specifically designed to target chAnxA6 for knockdown in DF-1 cells. The negative control siRNA (Catalog No. 12935–400) and transfection reagents were obtained from Invitrogen. For AnxA6 gene silencing, DF-1 cells at low passage were cultured in 35 mm dishes until they reached 80% confluency and then transfected with siRNA (10 μM) at a concentration of 30 pmol. The efficiency of knockdown was evaluated via immunoblot analysis 24 h post-transfection. To verify the specificity of AnxA6 knockdown, a rescue independent pDsRed-AnxA6 expression plasmid transfection was used in parallel validation experiments to confirm that the observed phenotypic changes were attributable to AnxA6 depletion rather than off-target effects. To examine the impact of AnxA6 knockdown on NDV replication, DF-1 cells treated with AnxA6 siRNA#3, control siRNA, or non-transfected cells were infected with the Goose/China/ZJ1/2000 strain at an MOI of 0.1. Cell culture supernatants and cell pellets were collected at 12, 24, 48, and 60 h post-infection (hpi), and viral titers were determined using the 50% tissue culture infective dose (TCID_50_) assay in DF-1 cells, following the Reed and Muench method [50]. Relative protein levels were quantified using densitometry analysis with ImageJ software (version 1.8.0), normalized to the expression of the control protein β-actin.

### 2.7. AnxA6 Overexpression and Virus Infection

To assess the effect of AnxA6 overexpression on NDV replication, DF-1 cells were transfected with constructed plasmids or empty vector controls to express DsRed-AnxA6 or DsRed. Following transfection, the cells were infected with NDV at a multiplicity of infection (MOI) of 0.1. At 24 h post-infection (hpi), hemagglutination (HA) titers and mRNA expression levels of the NDV M gene were measured in both culture -supernatants and cell pellets. The relative expression levels of the NDV M gene in AnxA6-overexpressing cells were quantified and compared to those in non-transfected cells after NDV infection. Cell viability was monitored in parallel using a CCK-8 assay to confirm that observed changes in viral replication were not attributable to differences in cell number or viability caused by AnxA6 overexpression or DsRed transfection.

### 2.8. Real-Time Quantitative PCR Analysis of M Gene mRNA

To measure NDV M mRNA levels, SYBR green-based real-time PCR (TaKaRa, Shiga, Japan) was utilized, with GAPDH mRNA employed as an internal control to normalize total RNA concentrations across samples. Total RNA was reverse-transcribed using oligo(dT) primers and M-MLV reverse transcriptase (TaKaRa, Shiga, Japan) according to the manufacturer’s protocol. The primers M-F (5′-GCTTGTAAGGCGAGAGGTG-3′) and M-R (5′-AACCTGGGGAGAGGCATTTG-3′) were designed to amplify the M gene, while G-F (5′-ATCACAGCCACACAGAAGACGG-3′) and G-R (5′-CTTTCCCCACAGCCTTAGCAGC-3′) were used to amplify GAPDH. RNA extraction and real-time PCR were carried out following a previously described protocol [17]. The standard curve method was applied to determine the fold-change in M gene mRNA expression levels. The M gene-specific primers were designed to target a conserved region of the NDV M gene coding sequence, with the amplicon verified by agarose gel electrophoresis to confirm a single product of the expected size. Amplification efficiency was determined from the standard curve and was >95% for both M gene and GAPDH assays. For extracellular (supernatant) samples, total RNA was extracted from clarified culture supernatants using the same RNA extraction protocol, and the presence of cell-free viral RNA was confirmed by the appearance of a single amplification peak in the melting curve analysis. The use of GAPDH as the internal reference gene was validated by confirming comparable Ct values across all experimental conditions. For supernatant samples, GAPDH was used as the normalization reference because NDV virulent infection induces severe cytopathic effect (CPE) with extensive cell lysis, resulting in the release of intracellular GAPDH into the culture supernatant. The comparable GAPDH Ct values across experimental conditions in supernatant samples confirmed its suitability as a reference for these samples. All RT-qPCR reactions were performed in technical triplicate, and results are reported as the mean of biological triplicates.

### 2.9. Statistical Analysis

Statistical analysis of differences in gene and protein expression levels, as well as viral titers, was performed using SPSS software (version 12.0; SPSS Inc., Chicago, IL, USA) [3]. Data were evaluated using an independent sample *t*-test for pairwise comparisons, with one-way ANOVA followed by Tukey’s post hoc test for multi-group comparisons. All experiments were performed with at least three independent biological replicates, and technical triplicates were used for each biological replicate where applicable. Results are presented as mean ± standard deviation (SD). A *p*-value of less than 0.05 was considered statistically significant. Levels of statistical significance are indicated by asterisks (* *p* < 0.05, ** *p* < 0.01, *** *p* < 0.001).

## 3. Results

### 3.1. Intracellular Localization of M and AnxA6

**Figure 1:**
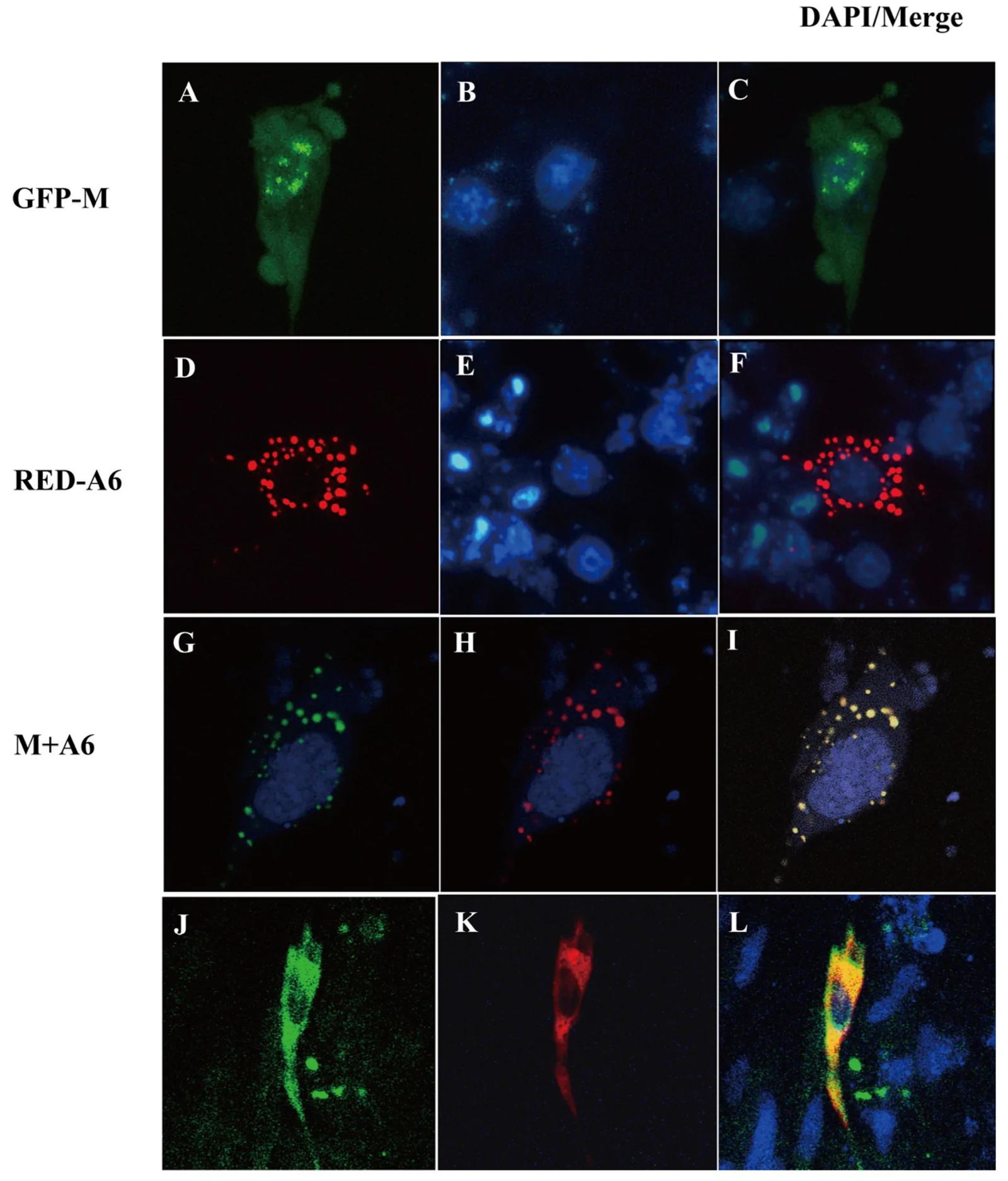
Intracellular distribution of M and AnxA6. (**A**–**C**) HEK-293T cells were transiently transfected with the plasmid expressing GFP-tagged M; GFP-M showed predominantly nuclear and nucleolar localization. (**D**–**F**) HEK-293T cells were transiently transfected with the plasmid expressing DsRed-tagged AnxA6; AnxA6 was primarily localized in the cytoplasm. (**G**–**I**) HEK-293T cells were co-transfected with both GFP-tagged M and DsRed-tagged AnxA6 plasmids. Upon co-expression, GFP-M nuclear accumulation was reduced, and M protein co-localized with AnxA6 predominantly in the cytoplasm near the nuclear membrane, as indicated by merged yellow signals. (**J**–**L**) DF-1 cells were transfected with Myc-tagged AnxA6 and then infected with NDV at an MOI of 0.1, fixed at 16 h post-infection, and stained with anti-M (rabbit) and anti-Myc (mouse) primary antibodies followed by Alexa Fluor 488-conjugated and Alexa Fluor 594-conjugated secondary antibodies. M protein and AnxA6 co-localized in the cytoplasm of infected cells. DAPI was used to stain nuclei. Scale bars, 20 μm. Representative images from at least three independent experiments are shown.

The M protein interacted with the AnxA6 protein, suggesting its co-localization within the cell. Initially, we cotransfected pEGFP-M and pDsRed-AnxA6 into HEK-293T cells and observed that GFP-M was predominantly localized in the nucleus and nucleolus (Figure 1A–C), whereas pDsRed-AnxA6 was primarily localized in the cytoplasm (Figure 1D–F). However, upon co-transfection, the M and AnxA6 proteins exhibited evident co-localization throughout the entire cell, primarily in the cytoplasm near the nuclear membrane (Figure 1G–I). Notably, when GFP-M was expressed alone, a strong nuclear and nucleolar accumulation was observed, which was reduced upon co-expression with AnxA6, suggesting that AnxA6 may influence the subcellular distribution of the M protein.

To further investigate their co-localization in virus-infected cells, we fixed cells at 16 h post-infection in DF-1 cells and determined the localization of the M and AnxA6 proteins using indirect immunofluorescence. The results demonstrated that in virus-infected cells, M protein and AnxA6 protein co-localized in the cytoplasm (Figure 1J–L). These findings were consistent with the fluorescence patterns observed in transient transfection plasmids (Figure 1I). The experimental outcomes indicated that during viral infection or plasmid co-transfection, the M protein and AnxA6 protein exhibited clear co-localization in the cytoplasm.

### 3.2. M Protein Interacted with Chicken AnxA6

**Figure 2:**
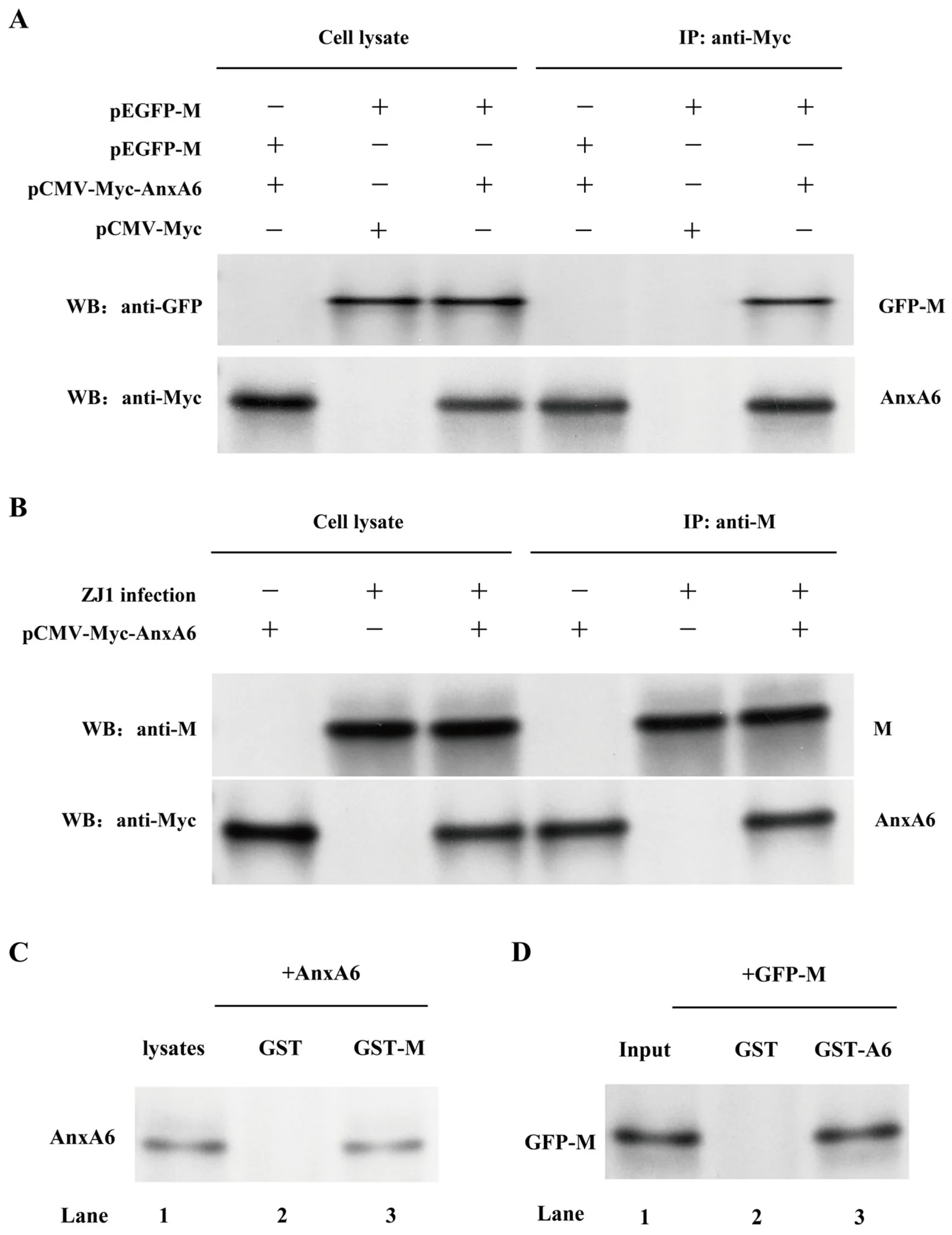
Interaction between M and AnxA6 in vitro and in vivo. (**A**,**B**) The interaction of M and AnxA6 was examined by reciprocal co-immunoprecipitation assay in DF-1 cells. (**C**,**D**) GST Pull-down assay of GST-AnxA6 or GST-M interaction.

We further confirmed the interaction between the M protein and AnxA6 by co-immunoprecipitation and GST pull-down assays. Co-immunoprecipitation results indicated that the co-expression of pEGFP-M and pCMV-Myc-AnxA6 in DF-1 cells resulted in detectable expression of both proteins when anti-GFP or anti-Myc antibodies were used. Specifically, co-immunoprecipitation with an anti-Myc antibody, followed by detection with an anti-GFP antibody, revealed the presence of the M protein bound to AnxA6, suggesting an in vivo interaction between the two proteins (Figure 2A).

Because of the limitations of accurately portraying the interactions between these proteins through exogenous transient expression methods, we attempted to replicate the natural interaction state of the M protein and AnxA6 by transfecting AnxA6 into DF-1 cells before infecting them with NDV. Subsequent detection using anti-M polyclonal antibodies and compatible labeled antibodies illustrated the expression of the M protein in NDV-infected cells and AnxA6 in cells transfected with pCMV-Myc-AnxA6. Immunoprecipitation with an anti-M antibody followed by detection with an anti-Myc antibody in NDV-infected cells revealed the expression of AnxA6, confirming the natural interaction between the M protein and AnxA6 (Figure 2B).

Moreover, GST pull-down assays demonstrated that the GST-M fusion protein selectively interacted with AnxA6 in DF-1 cell lysates, whereas the GST-AnxA6 fusion protein specifically bound to GFP-M in HEK-293T cell lysates transfected with pEGFP-M. Interestingly, the GST did not exhibit any binding interactions with AnxA6 or GFP-M, providing further evidence of the interaction between the M and AnxA6 proteins in vitro (Figure 2C,D).

### 3.3. Interfering with AnxA6 Did Not Affect the Virus’s Early Entry into Cells

**Figure 3:**
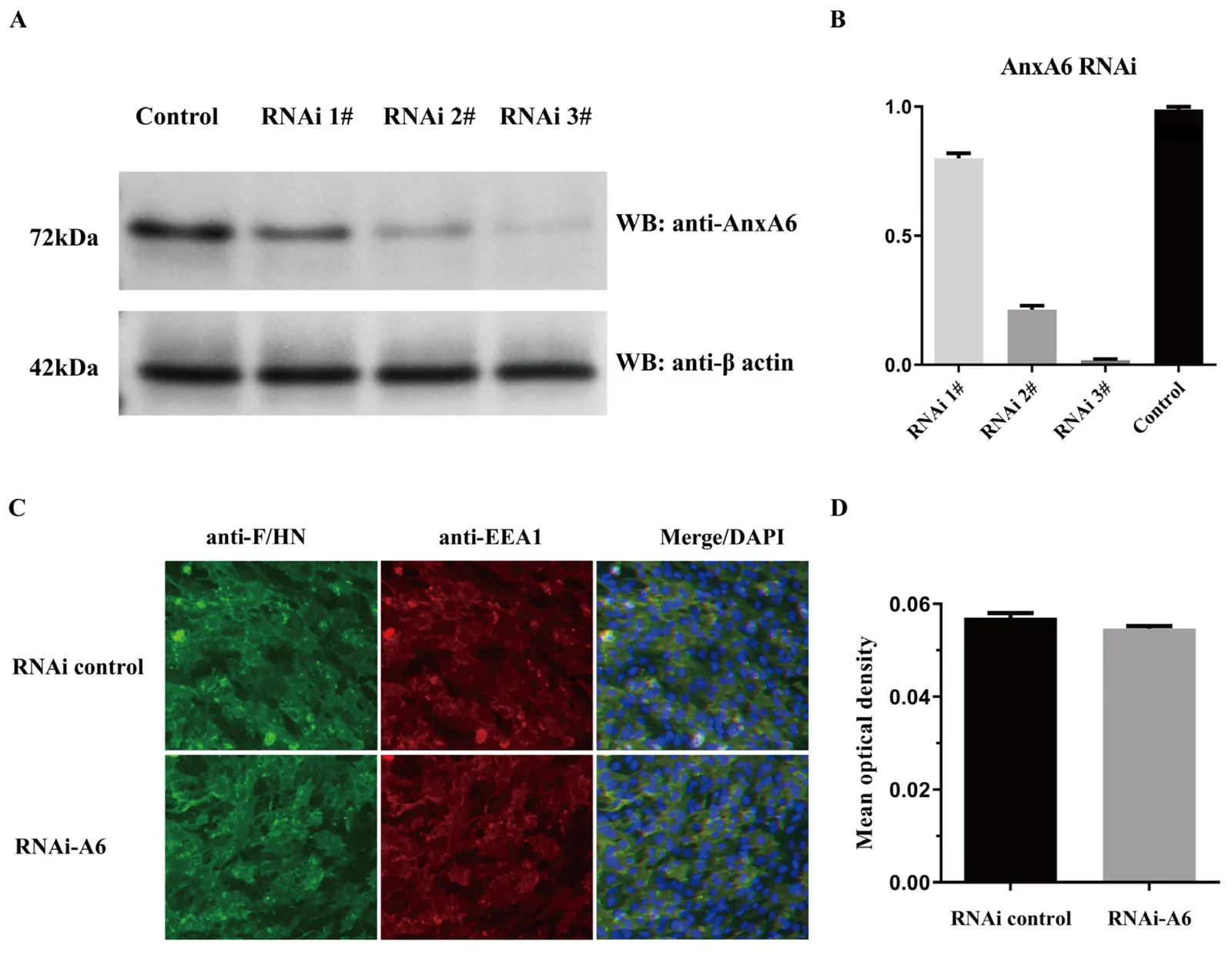
AnxA6 depletion does not affect virus entry into cells. (**A**) Western blot showing the effects of AnxA6 RNAi on the expression of endogenous AnxA6 protein in DF-1 cells. Molecular weight markers are indicated on the left. β-actin was used as a loading control. (**B**) Quantification of AnxA6 protein levels from panel (**A**) by densitometry. The density of bands was quantitated using ImageJ software and normalized to β-actin. Data represent mean ± SD from three independent experiments. (**C**) Immunofluorescence analysis of the effects of AnxA6 depletion on virion entry. DF-1 cells transfected with control siRNA or AnxA6 siRNA were infected with NDV (MOI = 0.1) and fixed at 4 hpi. F/HN protein (green) and EEA1 (red) were detected by immunofluorescence. Nuclei were stained with DAPI. No significant difference in F/HN fluorescence intensity or EEA1 co-localization was observed between control and AnxA6-depleted cells, indicating that AnxA6 depletion did not significantly alter early viral entry. (**D**) Mean optical density of fluorescence in control siRNA- or AnxA6 siRNA-treated DF-1 cells from panel (**C**). Data represent mean ± SD from three independent experiments. Scale bars, 20 μm.

DF-1 cells were separately transfected with the three pairs of synthesized AnxA6 siRNAs and control siRNA, and Western blot was conducted to assess the impact on AnxA6 protein expression 24 h after transfection. The findings revealed that AnxA6 RNAi#3 exhibited the most potent inhibitory effect on AnxA6 protein expression (Figure 3A,B).

Next, AnxA6 siRNA (RNAi#3) and control siRNA were individually reintroduced into DF-1 cells and infected with NDV 24 h post-transfection. The cells were fixed after 4 h of infection, and indirect immunofluorescence was used to monitor the expression of the F/HN protein and the early endosome-associated protein EEA1. The results demonstrated that despite interference with AnxA6 protein expression, NDV replicated efficiently within the cells (Figure 3C,D). These experimental results indicated that AnxA6 depletion did not significantly alter the early entry of NDV into DF-1 cells under the conditions tested. However, because the immunofluorescence signal at 4 hpi reflects both internalized and replicating virus, these data do not exclude a role for AnxA6 in later stages of the viral entry pathway, such as endosomal trafficking or membrane fusion.

### 3.4. Knockdown of AnxA6 by siRNA Promoted NDV Growth in DF-1 Cells

**Figure 4:**
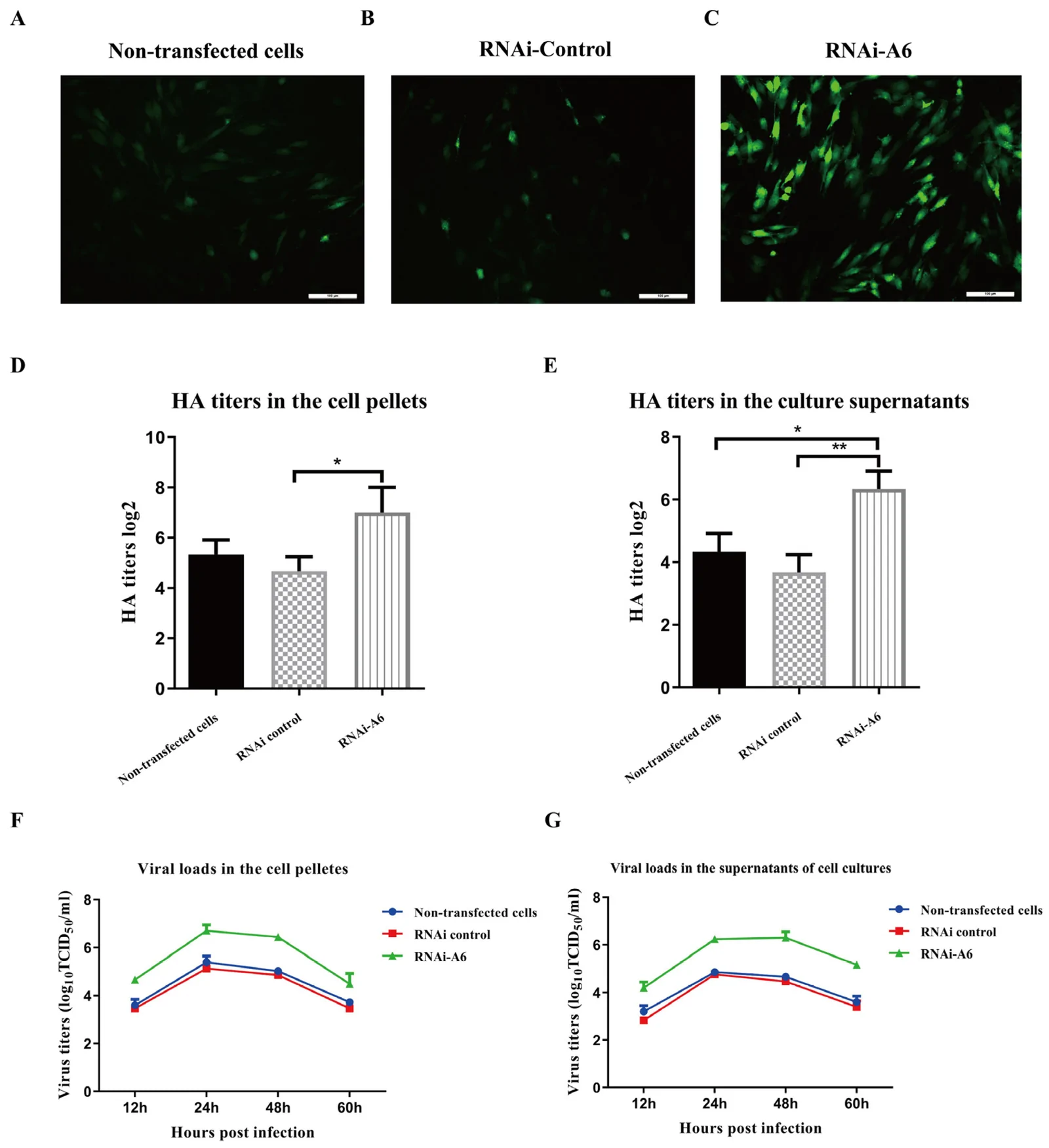
Knockdown of AnxA6 promotes NDV replication in DF-1 cells. (**A**–**C**) Morphological changes and GFP fluorescence of DF-1 cells after NDV (rZJ1GFP) infection. Non-transfected cells, RNAi control cells, and RNAi-AnxA6 cells were infected with NDV at an MOI of 0.1, and images were captured at 24 hpi. Enhanced GFP fluorescence indicates increased viral spread. Bright-field images show corresponding cell morphology. Scale bars, 100 μm. (**D**,**E**) Quantitative analysis of NDV HA titers in cell pellets (**D**) and culture supernatants (**E**) at 24 h post-infection. (**F**,**G**) Knockdown of AnxA6 induced NDV growth. At different time points (12, 24, 48 and 60 hpi), the viral loads in the cell pellets (**F**) and cell culture supernatants (**G**) were determined by TCID_50_ assay in DF-1 cells. The significance of the difference between RNAi-AnxA6 and RNAi control treatments was determined using one-way ANOVA followed by Tukey’s post hoc test (** *p* < 0.01 and * *p* < 0.05). Data represent mean ± SD from three independent experiments.

To understand the impact of disrupting AnxA6 protein expression on viral replication, DF-1 cells were separately transfected with AnxA6 siRNA and control siRNA, followed by infection with the ZJ1 virus 36 h post-transfection. After 24 h of viral infection in the cells, inhibition of AnxA6 protein expression markedly increased the cytopathic effects induced by the virus within the cells, as visualized by the enhanced GFP fluorescence emitted by the recombinant NDV strain rZJ1GFP, which constitutively expresses green fluorescent protein as a reporter of viral infection and spread. In contrast, the RNAi control group and non-transfected cells exhibited milder cytopathic effects following viral infection than the AnxA6 interference group (Figure 4A–C). This suggested that the interference with AnxA6 protein expression enhanced viral replication.

Virus hemagglutination (HA) titers in the cell precipitate and cell supernatant 24 h post-viral infection revealed elevated virus HA titers in the AnxA6-RNAi group relative to those in the RNAi control group and the non-transfected group (Figure 4D,E), aligning with the cytopathic effects observed at 24 h post-infection. Further examination of virus loads results at various time points post-infection demonstrated notably higher viral titers in both the cell precipitate and cell supernatant of the AnxA6 interference group compared to the RNAi control group and non-transfected cells (*p* < 0.01). However, virus titers peaked at 24 h post-viral infection across all groups (Figure 4F,G). This indicated that disrupting AnxA6 protein expression can facilitate NDV replication.

### 3.5. Overexpression of AnxA6 Inhibited NDV Replication in DF-1 Cells

**Figure 5:**
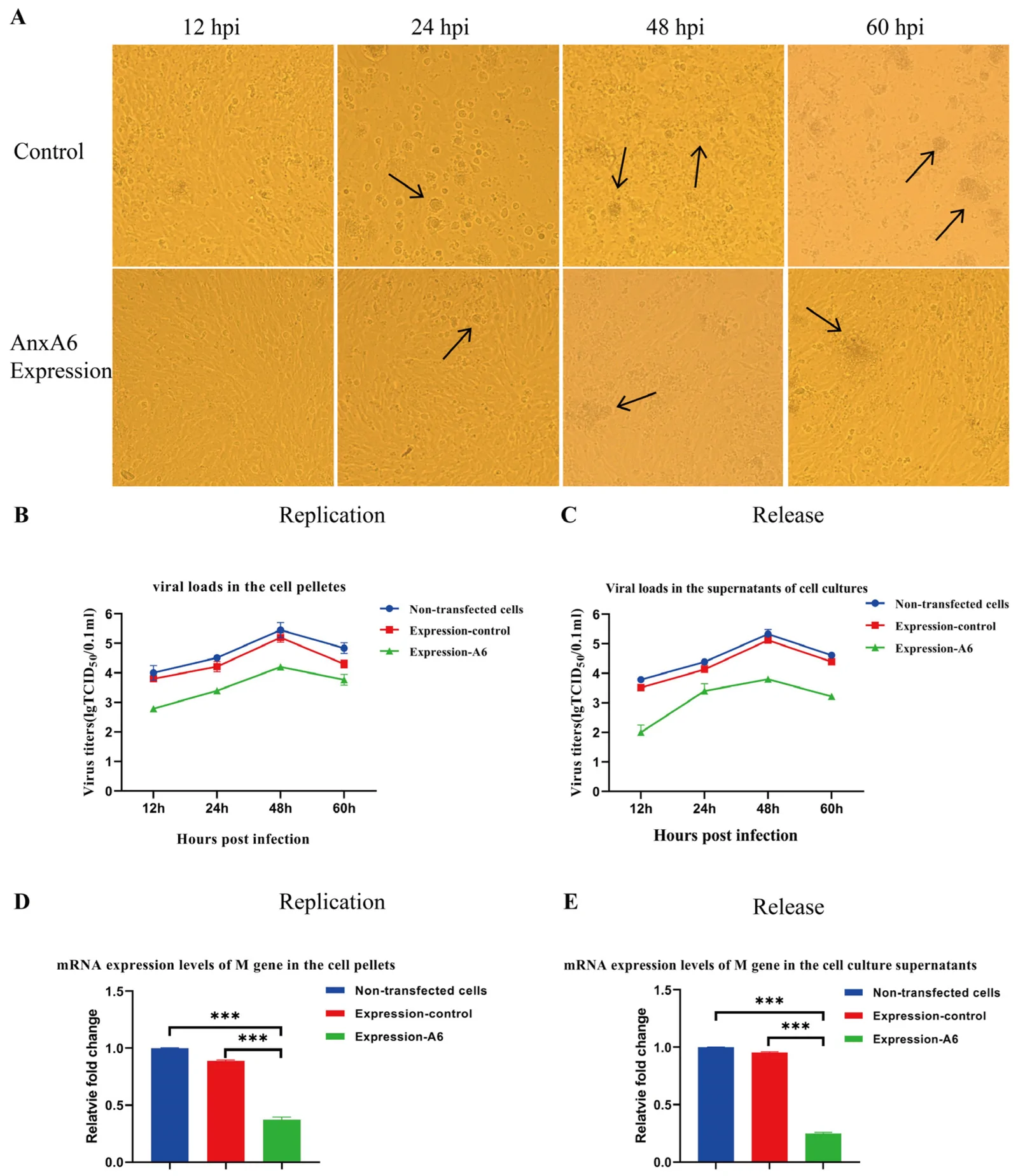
AnxA6 overexpression inhibits NDV replication. (**A**) Morphological changes and GFP fluorescence of DF-1 cells during NDV (rZJ1GFP) infection. DsRed (control) or DsRed-AnxA6-overexpressing cells were infected with NDV at an MOI of 0.1, and images were captured at 12, 24, 48 and 60 hpi. Scale bars, 100 μm. The arrow pointed to the cytopathic effect. (**B**,**C**) Overexpression of AnxA6 reduced NDV growth. TCID_50_ titers in cell pellets (**B**) and supernatants (**C**) at 12, 24, 48 and 60 hpi. (**D**,**E**) Real-time RT-PCR determination of NDV M gene mRNA expression levels. “Non-transfected cells” refers to untransfected DF-1 cells infected with NDV (set as 100%); “Expression-control” refers to empty vector-transfected cells infected with NDV; “Expression-A6” refers to AnxA6-overexpressing cells infected with NDV. M gene mRNA levels were normalized to the internal reference gene GAPDH, and relative expression was calculated as the ratio to the non-transfected group. Data represent mean ± SD from three independent experiments. The significance of the difference was determined by one-way ANOVA followed by Tukey’s post hoc test (*** *p* < 0.001).

We conducted a more in-depth investigation into the impact of AnxA6 on NDV replication by increasing the expression of AnxA6 protein within cells. Initially, we elevated the expression levels of both pDsRed-C1 and pDsRed-AnxA6 in DF-1 cells, which were then infected with the ZJ1 virulent virus at 12, 24, 48, and 60 h post-inoculation. Assessment of the cytopathic effect (CPE) of NDV in DF-1 cells subsequent to protein overexpression indicated that the heightened expression of pDsRed-AnxA6 notably diminished the cytopathic effects induced by NDV on the cells, whereas the control group, pDsRed-C1, did not affect the virulence of NDV in the cells (Figure 5A).

Virus titration of the cellular precipitate and supernatant revealed a significant influence of pDsRed-AnxA6 overexpression on virus production within the cells (Figure 5B,C). Moreover, fluorescence quantitative PCR analysis of viral M gene expression levels in the cell precipitate and supernatant post-infection showed that the overexpression of pDsRed-AnxA6 significantly reduced viral M mRNA levels in both cell-associated and supernatant fractions compared with the empty vector (DsRed) control (Figure 5D,E). The relative M gene expression values shown in Figure 5D,E represent the ratio of M gene copies (normalized to GAPDH) in each experimental group relative to the non-transfected (untransfected, NDV-infected) control group, which was set as 100%. These results indicated that the overexpression of AnxA6, which interacted with the M protein inside cells, inhibited NDV replication.

### 3.6. C-Terminal of M Protein Dominated in M-AnxA6 Interaction

**Figure 6:**
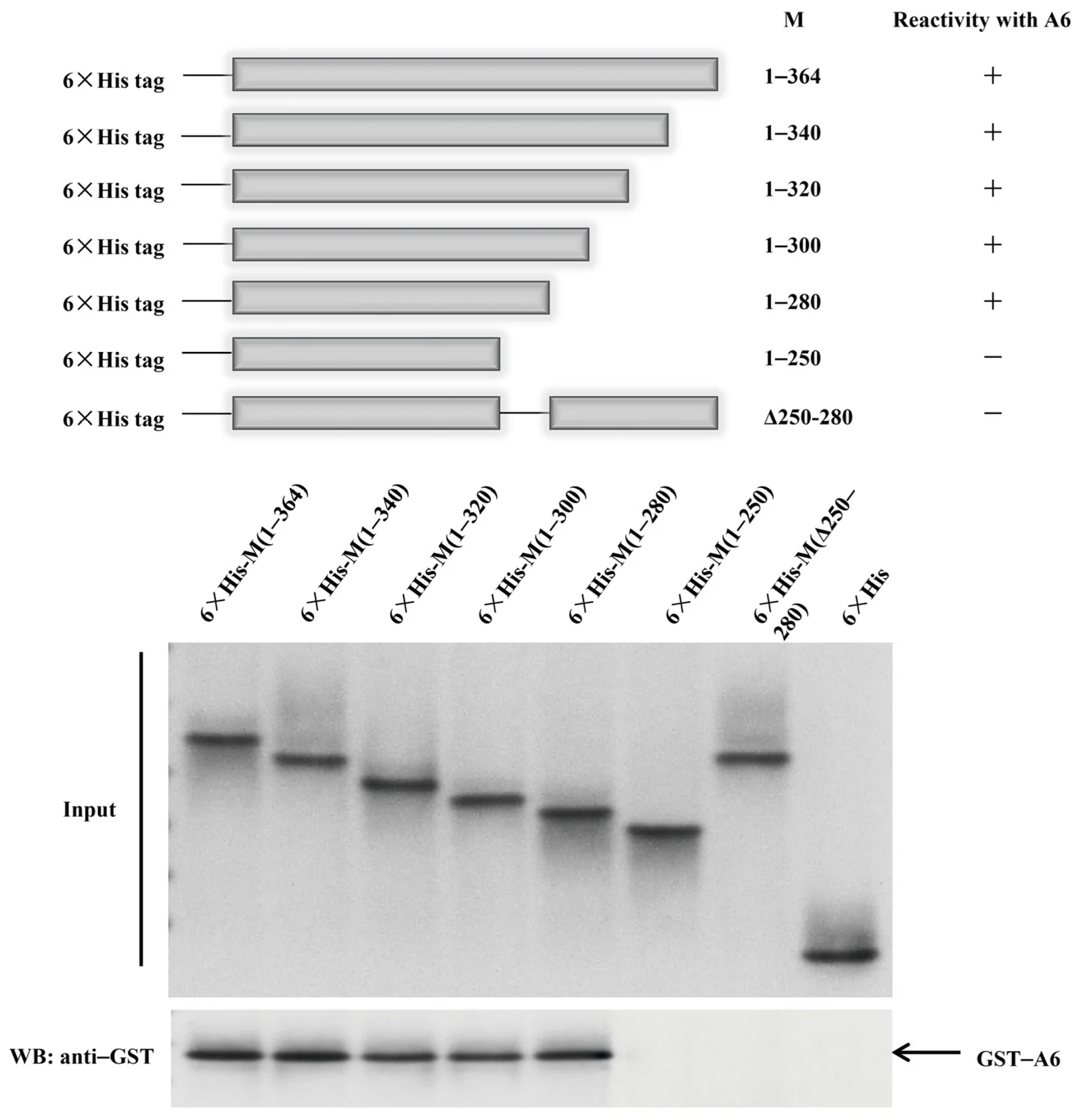
Mapping binding domains between M and AnxA6. His pull-down mapping domains of M for AnxA6 binding. Upper panel, schematic representation of full-length and deletion mutants of 6×His-tagged M (6His-M). The M protein amino acid numbering is based on the ZJ1 strain sequence (GenBank: AF431744). The region aa 250–280 lies within the C-terminal domain of the M protein, which is predicted to be involved in membrane association and protein-protein interactions based on structural modeling of paramyxovirus matrix proteins. Upper panel, various purified 6His-M or its deletion mutants as indicated were incubated with GST-AnxA6 protein and the pulled-down proteins were immunoblotted anti-His (upper) or anti-GST (lower). The integrity and equal loading of all His-tagged constructs were confirmed by anti-GST Western blotting (lower panel). Results are representative of three independent experiments.

Using His pull-down assays, we further characterized the domains responsible for the interaction between M and AnxA6 proteins. Results revealed that the fusion proteins 6 His-M (1–250) and 6 His-M (Δ250–280) did not bind to AnxA6, whereas the fusion protein containing M (250–280), 6 His-M, interacted with AnxA6 (Figure 6). This suggested that the region of aa 250–280 in the M protein was crucial for binding with AnxA6. This region corresponds to the C-terminal portion of the NDV M protein, which has been implicated in membrane association and protein-protein interactions important for viral assembly. The integrity and equal loading of all His-tagged M protein constructs used in the pull-down assay were confirmed by anti-His Western blotting, and the results are consistent across three independent experiments. It should be noted that these mapping experiments identify a region necessary for binding but do not exclude the contribution of adjacent residues; future structural studies will be needed to precisely define the interaction interface.

## 4. Discussion

M and M-like proteins play vital roles in the replication of enveloped viruses, specifically in the viral assembly and budding processes [6,7,13]. These proteins are dependent on the host cell machinery owing to the inherent replication constraints of the virus, effectively leveraging cellular components to facilitate efficient viral replication [14,15,16]. Several studies demonstrated the ability of RNA viruses to exploit host cells for propagation [1,4,5]. For example, interactions between the M proteins of the NDV and Sendai virus with cellular actin have been documented to promote virus budding [6,26]. Further exploration of the budding mechanism of the Sendai virus M protein revealed that the late YLDL domain of the M protein binds to the host cell protein Alix/Aip1, thereby facilitating viral replication and budding [6,14,15]. Recent findings have revealed the compensatory role of the host cell protein Alix for the Ebola virus VP40 protein in cases of late-domain damage induced by budding injury [14,16]. Additionally, interactions between the vesicular stomatitis virus (VSV) M protein and the host proteins mRNA export factor 1 (Rae1) and nucleoporin 98 (Nup98) have been identified, potentially influencing the nuclear translocation mechanism of the M protein [4,9].

A yeast two-hybrid approach identified angiomotin-like 1 and 14–3–3 as host cell proteins that interact with human parainfluenza virus type 5 (hPIV5) M protein [51,52]. Angiomotin-like 1 promotes the release of virus-like particles (VLPs) to enhance viral budding, whereas 14–3–3 inhibits virus budding, thereby reducing viral replication efficiency [51,52,53]. Immunoprecipitation is an effective method for studying protein interactions. Studies have utilized immunoprecipitation to capture proteins interacting with human respiratory syncytial virus (HRSV) M proteins, highlighting proteins such as B23 and F-actin that are capable of interacting with the M protein and impacting viral replication [4,23,24,25]. Despite the low homology among M proteins across paramyxoviruses, host proteins such as AP3B1 have been reported to interact with Nipah virus and Hendra virus M proteins, leading to inhibition of viral replication [7,13]. Collectively, these studies underscore the pivotal role of host proteins in promoting or hindering viral replication, underscoring their significance in viral pathogenesis [17,18,19,20].

The critical involvement of the M protein in the infection and replication of paramyxoviruses, exemplified by NDV as a member of the paramyxovirus family, has been a focus for investigating the interactions between host proteins and the M protein [1,4,5]. Early reports have indicated interactions between NDV M proteins and host cell actin [6,26]. Yeast two-hybrid methods have been used to detect the interaction between the host protein CHMP4 and the NDV M protein, with subsequent investigations revealing its -facilitative role in NDV replication [21,26]. Furthermore, research has confirmed that the interaction between the host nuclear phosphoprotein B23 and the NDV M protein influences virus replication through the modulation of M protein localization in the nucleolus [4,23,24]. The mechanism of nuclear and nucleolar localization of the NDV M protein facilitates subverting host genetic information and impeding the transcription and translation of host genes to support viral replication [4,8,9].

Annexins are a class of proteins expressed in most eukaryotic cells that bind to the host cell membrane and exhibit diverse functions including vesicle transport, cell division, apoptosis, calcium signaling, and growth regulation [36,37,38]. Annexin A6 (AnxA6), a distinct member of the annexin family, participates in the Ca^2+^ signaling pathway owing to its specific structural attributes [34,39]. Studies have indicated that, during influenza virus infection, AnxA6 interacts with the μ1 and μ2 subunits of the AP1-clathrin complex involved in endocytosis, thereby influencing virus budding and negatively regulating viral infections [33,40,41]. Proteomic analysis of NDV infection revealed significant alterations in AnxA6 expression, suggesting its potential role in NDV infection [30,31]. Experimental observations in HEK-293T and DF1 cells infected with NDV confirmed the interactions between M protein and AnxA6, supporting the proposed interaction. Functional studies involving RNAi and AnxA6 overexpression in host cells have illustrated AnxA6’s negative regulatory impact on NDV replication, suggesting its potential as a therapeutic target.

Furthermore, the interaction between the M protein and AnxA6 was identified, specifying the critical region of the M protein aa250–280 binding to the AnxA6 protein. Research suggests that the carboxy-terminus of the NDV M protein is indispensable for its function and contributes significantly to viral assembly and budding [43,50]. The interaction between the M protein and AnxA6, mapped to the aa250–280 region, underscores the importance of this C-terminal segment in the M-AnxA6 interaction. Although our deletion mapping experiments localize the binding region to aa 250–280, the present data do not establish whether an isolated peptide corresponding to this region is sufficient for binding, and further mutagenesis and structural studies are needed to precisely delineate the interaction interface. Future investigations should further investigate the intricate mechanisms governing AnxA6-M protein interactions.

The ESCRT machinery plays a crucial role in viral budding across multiple virus families, including paramyxoviruses [14,15,16]. Our findings suggest that AnxA6 may modulate NDV replication through interactions with the M protein, potentially affecting viral assembly and/or release. However, the present data do not distinguish whether the M-AnxA6 interaction directly mediates the observed effects on viral replication, or whether AnxA6 acts through parallel pathways that indirectly influence viral yield. Further experiments, such as targeted mutagenesis of the M protein interaction interface and rescue of mutant viruses, will be needed to establish a direct mechanistic link. This mechanism is reminiscent of the interaction between influenza A virus M2 protein and AnxA6, where AnxA6 was shown to negatively regulate viral budding [33,39,42]. The conservation of this regulatory mechanism across different virus families highlights the importance of host factors in viral life cycles.

Advances in structural biology have provided insights into the organization of paramyxovirus matrix proteins [1,43]. Cryo-EM studies have revealed that the M protein forms a lattice-like structure beneath the viral envelope, which is essential for viral assembly [1]. The C-terminal domain of the M protein, where AnxA6 binds, may play a role in organizing this structure or in interactions with host cell membranes during viral budding [7,13]. Understanding the structural basis of the AnxA6-M interaction will be crucial for developing antiviral strategies targeting this host-virus interface.

The innate immune response to NDV infection involves multiple signaling pathways, including the RIG-I pathway [28,29]. While our study focused on the direct interaction between AnxA6 and the M protein, it is possible that AnxA6 may also modulate NDV replication through effects on host immune responses. Further studies are needed to investigate whether AnxA6 affects NDV replication through interferon-dependent or independent mechanisms. Additionally, the role of autophagy in NDV replication has been documented [44,45], and it would be interesting to determine whether AnxA6 interacts with autophagic pathways during NDV infection.

## 5. Conclusions

In summary, this study investigated the interaction between NDV M and host AnxA6 proteins, suggesting that AnxA6 is associated with reduced NDV replication in this in vitro cell culture model. While affirming the importance of this interaction, further studies using proteomics and advanced technologies are imperative to comprehensively decipher the implications of AnxA6 in NDV infection in cell culture. The discovery of this host-virus interaction provides new insights into NDV biology and highlights AnxA6 as a potential target for future antiviral investigation, although extrapolation to in vivo pathogenesis and therapeutic application will require further validation.

Several limitations of this study should be acknowledged. First, the study was conducted in a single cell line (DF-1), and the results may not be generalizable to other cell types or in vivo systems. Second, the knockdown experiments relied primarily on a single siRNA, future studies employing additional independent siRNAs or CRISPR-based approaches would further strengthen the conclusions. Third, while the M-AnxA6 interaction was demonstrated by multiple assays, the present data do not establish that this interaction directly mediates the observed effects on viral replication, as opposed to AnxA6 acting through parallel pathways. Fourth, all observations are based on cultured cells, and claims regarding pathogenicity or virulence cannot be made solely from in vitro data. Fifth, the AnxA6 rescue experiment confirmed restoration of AnxA6 protein expression but did not demonstrate reversal of the viral replication phenotype; therefore, the functional specificity of the knockdown cannot be fully excluded based on the current data. Future studies using animal models and structural biology approaches will be essential to address these questions.

## Figures and Tables

**Table 1:** Primers used in this study.

Primer	Nucleotide Sequence (5′→3′) ^a^	Restriction Sites
GST-M/F	TGC*GAATTC*ATGGACTCATCCAG	*Eco*R I
GST-M/R	GGCA*CTCGAG*TTATTTCCTGAAAGG	*Xho* I
GFP-M/F	TCG*GAATTC*ATGGACTCATCCAG	*Eco*R I
GFP-M/R	GGCA*GTCGAC*TTATTTCCTGAAAGG	*Sal* I
Red-A6/F	AG*CTCGAG*ATGGCACCCAAAGGA	*Xho* I
Red-A6/R	TCGC*GAATT*CTAGTCGTCCCCCCCGC	*Eco*R I
GST-A6/F	ATCA*GGATCC*ATGGCACCCAAAGGA	*Bam*H I
GST-A6/R	ACG*CTCGAG*CTAGTCGTCCCCCCCGC	*Xho* I
Myc-A6/F	TG*GAATTC*CCATGGCACCCAAAGGA	*Eco*R I
Myc-A6/R	AG*CTCGAG*CTAGTCGTCCCCCCCGC	*Xho* I
His-M/F	TGC*GAATTC*ATGGACTCATCCAG	*Eco*R I
His-M/R	GGCA*GTCGAC*TTATTTCCTGAAAGG	*Sal* I

^a^ The restriction sites used in the primers were shown in italic.

**Table 2:** The sequences of siRNAs targeted the *AnxA6* gene.

siRNA	Sense (5′→3′)	Anti-Sense (5′→3′)
RNAi#1	CGGAUGAGAAGUGCCUCAUUGAGAU	AUCUCAAUGAGGCACUUCUCAUCCG
RNAi#2	GCAGCAGAUUCUGAAGGCCUACAAA	UUUGUAGGCCUUCAGAAUCUGCUGC
RNA#3	CGUGAAGAACAAGCCAGCCUUCUUU	AAAGAAGGCUGGCUUGUUCUUCACG

## Data Availability

The original contributions presented in this study are included in the article/Supplementary Material. Further inquiries can be directed to the corresponding authors.

## Supplementary Materials

The following supporting information can be downloaded at https://www.mdpi.com/article/10.3390/v18101164/s1. Figure S1. Amplification efficiency and melt curve for both M gene and GAPDH. Figure S2. To verify the specificity of AnxA6 knockdown, a rescue independent pDsRed-AnxA6 expression plasmid transfection was used to confirm that the observed phenotypic changes were attributable to AnxA6 depletion rather than off-target effects. Figure S3. Cell viability was monitored in parallel using a CCK-8 assay to confirm that observed changes in viral replication were not attributable to differences in cell number or viability caused by AnxA6 deletion or overexpression. Figure S4. The specificity of anti-body. Figure S5. Standard curve for both M gene and GAPDH. Figure S6. Fluorescence microscopy analysis confirmed that all truncation and deletion variants of the M protein were successfully expressed. Table S1. Primers used for truncated or deleted M.
