# Supplementary material for: Annexin A6 Interacts with Newcastle Disease Virus Matrix Protein and Is Associated with Reduced Viral Replication in DF-1 Cells
Source: Viruses. 2026 Oct 20;18(10):1164. doi: 10.3390/v18101164 (PMC13709843; doi:10.3390/v18101164)
Supplement: Supplementary file 1 [file viruses-18-01164-s001.zip › viruses-4571375-supplementary.pdf]

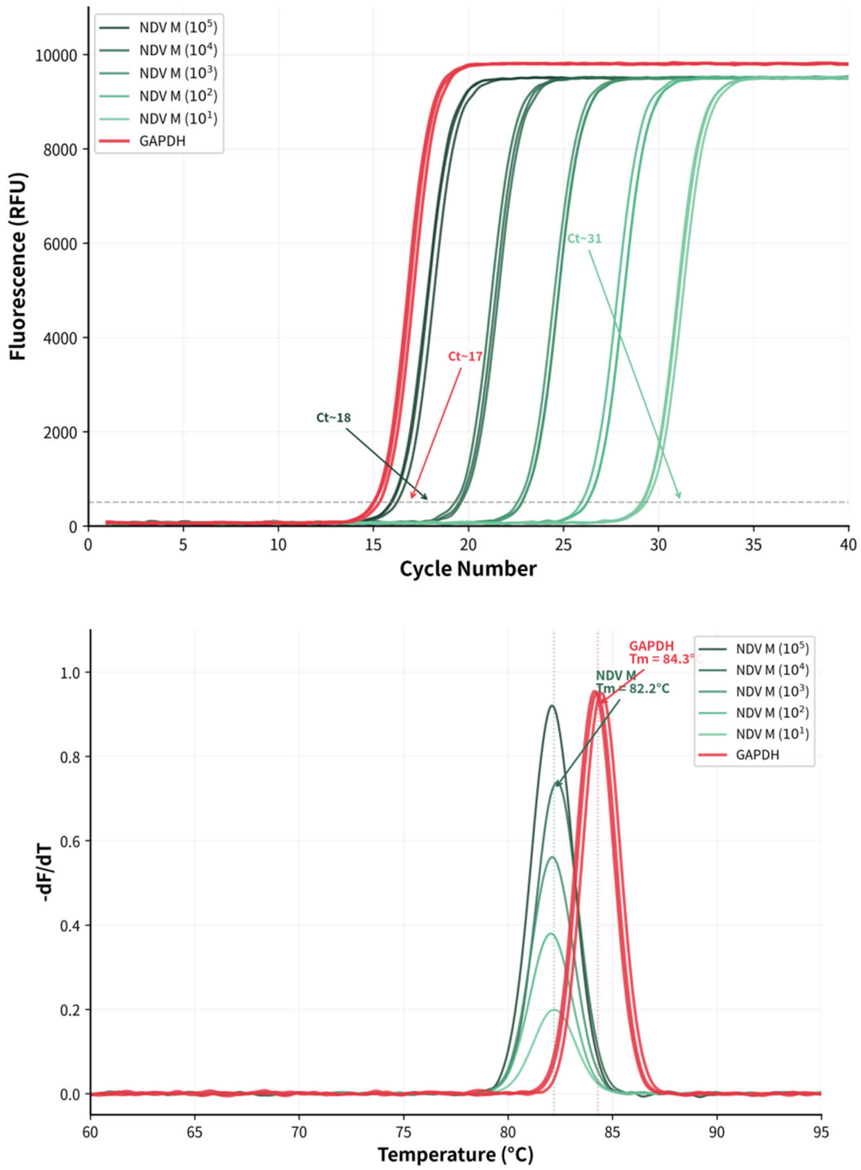

Figure S1. Amplification efficiency and melt curve for both M gene and GAPDH.

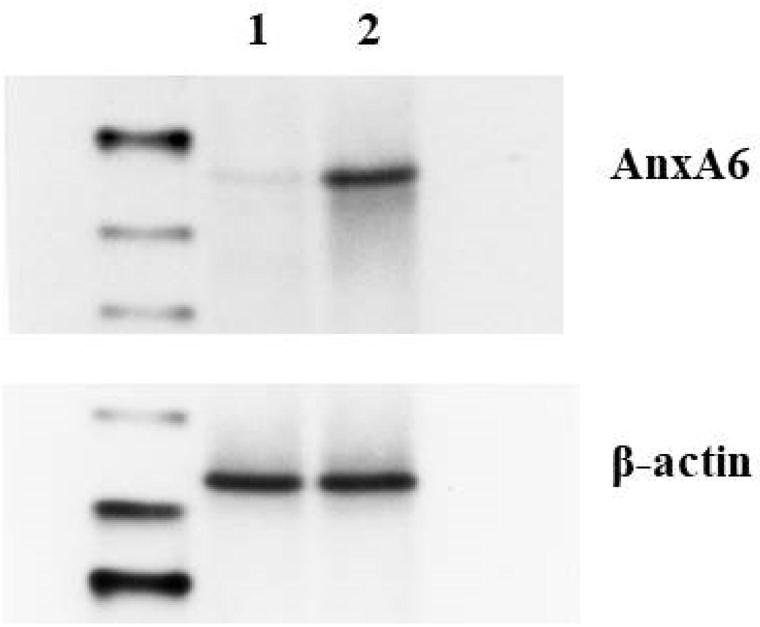

Figure S2. To verify the specificity of AnxA6 knockdown, a rescue independent pDsRed-AnxA6 expression plasmid transfection was used to confirm that the observed phenotypic changes were attributable to AnxA6 depletion rather than off-target effects. Lane 1 represent RNAi3#, Lane 2 represent RNAi3#+pDsRed-AnxA6.

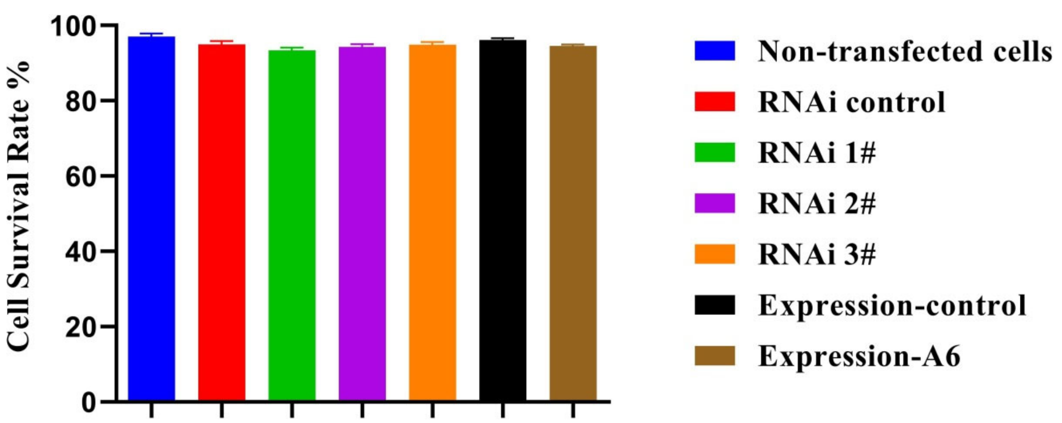

Figure S3. Cell viability was monitored in parallel using a CCK-8 assay to confirm that observed changes in viral replication were not attributable to differences in cell number or viability caused by AnxA6 deletion or overexpression.

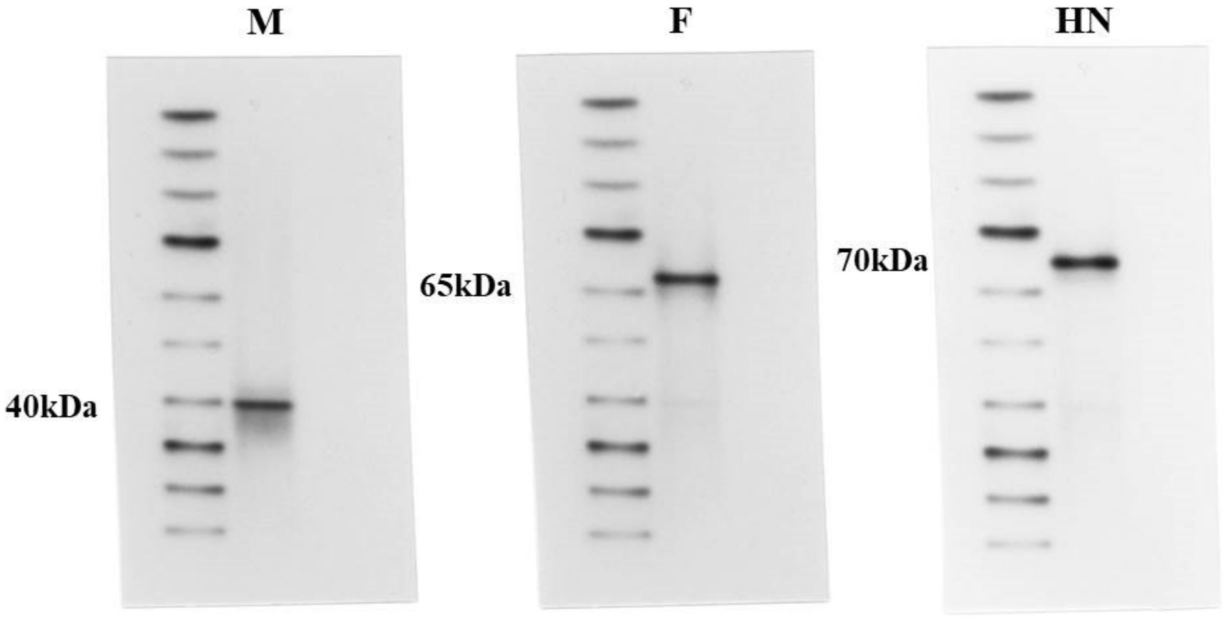

Figure S4. The specificity of anti-body.

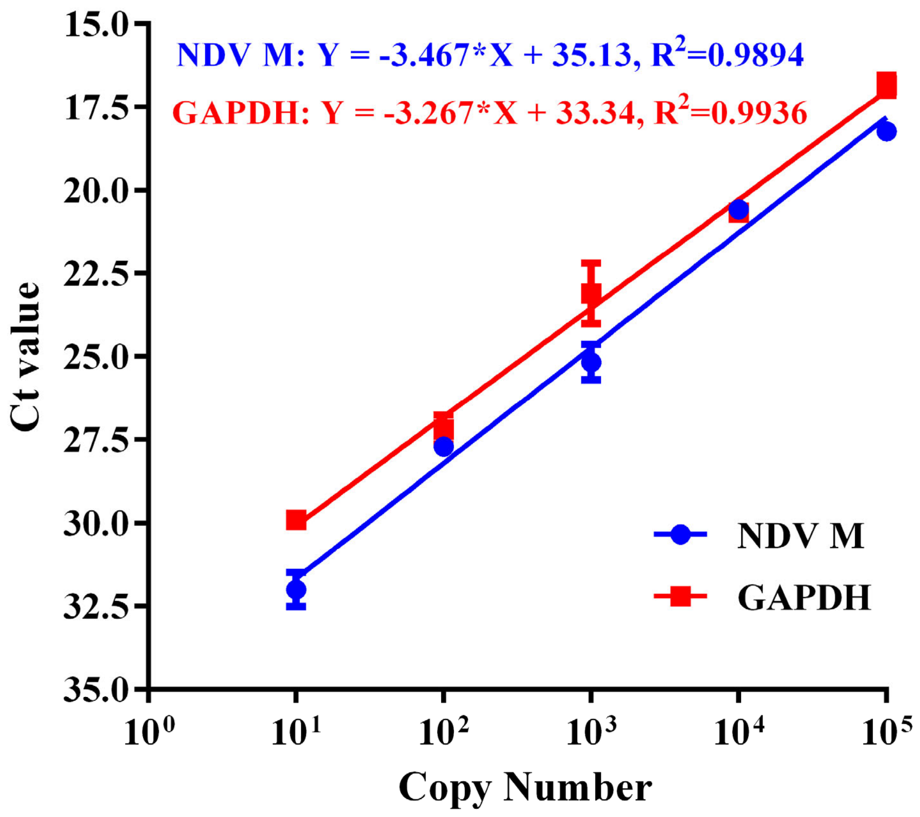

Figure S5. Standard curve for both M gene and GAPDH.

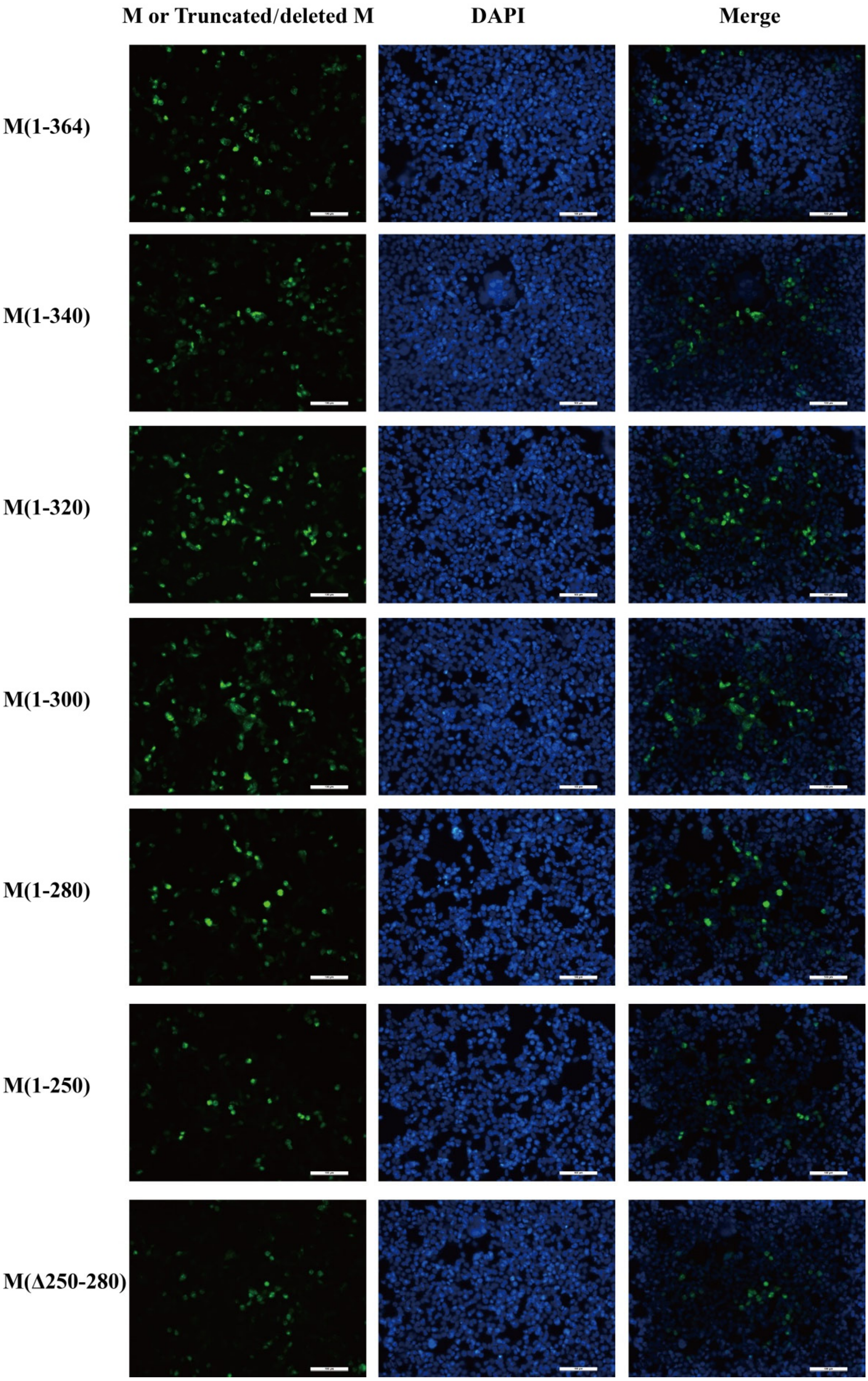

Figure S6. Fluorescence microscopy analysis confirmed that all truncation and deletion variants of the M protein were successfully expressed

Table S1. Primers used for truncated or deleted M

Primers name	Nucleotide (5’ to 3’)
EGFP-MF（EcoRI）	CGCGAATTCATGGACTCATCCAG
EGFP-MR-M(1-340)	GGGTCGACggTCACTGCGACAG
EGFP-MR-M(1-320)	GGGTCGACctCAGTTTGGCTCC
EGFP-MR-M(1-300)	GGGTCGACggCTGTCCCACTGC
EGFP-MR-M(1-280)	GGGTCGACcaCAAGCACAGAGG
EGFP-MR-M(1-250)	GGGTCGACctTTCCCTTCTTAT
EGFP-MR-M(280-364)	GGGTCGACtcCGCTATCGGACT
